# High-Throughput UAV Image-Based Method Is More Precise Than Manual Rating of Herbicide Tolerance

**DOI:** 10.34133/2019/6036453

**Published:** 2019-09-15

**Authors:** Hema S. N. Duddu, Eric N. Johnson, Christian J. Willenborg, Steven J. Shirtliffe

**Affiliations:** Department of Plant Sciences, College of Agriculture and Bioresources, University of Saskatchewan, 51 Campus Drive, Saskatoon, SK, Canada

## Abstract

The traditional visual rating system is labor-intensive, time-consuming, and prone to human error. Unmanned aerial vehicle (UAV) imagery-based vegetation indices (VI) have potential applications in high-throughput plant phenotyping. The study objective is to determine if UAV imagery provides accurate and consistent estimations of crop injury from herbicide application and its potential as an alternative to visual ratings. The study was conducted at the Kernen Crop Research Farm, University of Saskatchewan in 2016 and 2017. Fababean (*Vicia faba* L.) crop tolerance to nine herbicide tank mixtures was evaluated with 2 rates distributed in a randomized complete block design (RCBD) with 4 blocks. The trial was imaged using a multispectral camera with a ground sample distance (GSD) of 1.2 cm, one week after the treatment application. Visual ratings of growth reduction and physiological chlorosis were recorded simultaneously with imaging. The optimized soil-adjusted vegetation index (OSAVI) was calculated from the thresholded orthomosaics. The UAV-based vegetation index (OSAVI) produced more precise results compared to visual ratings for both years. The coefficient of variation (CV) of OSAVI was ~1% when compared to 18-43% for the visual ratings. Furthermore, Tukey's honestly significance difference (HSD) test yielded a more precise mean separation for the UAV-based vegetation index than visual ratings. The significant correlations between OSAVI and the visual ratings from the study suggest that undesirable variability associated with visual assessments can be minimized with the UAV-based approach. UAV-based imagery methods had greater precision than the visual-based ratings for crop herbicide damage. These methods have the potential to replace visual ratings and aid in screening crops for herbicide tolerance.

## 1. Introduction

Over the past decade, plant phenotyping has gained considerable attention, as more and more researchers realize that the lack of adequate phenotypic data is the bottleneck to achieve further genetic gains in plant breeding programs [1, 2]. Phenotype is the outcome of genotype and environment interaction, so the renewed interest in phenomics is also because of the ever-changing climate and the need for breeding more adaptable varieties [3]. This has resulted in an increased requirement for reliable and timely phenotypic data to support crop improvement programs [4, 5].

Traditional methods of measuring crop phenotypic characteristics relating to biotic/abiotic stress to crops are based on visual ratings or manual assessments [6, 7]. Although visual assessments are simple [8], there are associated disadvantages since they are labor-intensive, time-consuming, and prone to human error [9]. Furthermore, in breeding trials with hundreds or thousands of plots to rate, the greater time requirement with visual ratings increases the plant stage variability [7] and other issues related to inter/intra-rater differences may lead to inaccurate assessments [10].

Image-based remote-sensing tools such as manned aircrafts and satellites (conventional high-throughput platforms) have been used as an alternative approach as they offer rapid data gathering, consistency, and greater objectivity [11]. Since not all platforms have similar characteristics, a proper choice of systems and sensors based on the objective of the study is important for field phenotyping. For example, satellites can obtain image data over a large area in a short time; however, it may not be suitable for traits that require continuous monitoring because of their greater revisit cycles and vulnerability to environmental factors [12]. Some of the more recent high-throughput phenotyping platforms such as unmanned aerial vehicles (UAVs) offer a low-cost alternative capable of obtaining high spatial and temporal resolution imagery with greater operational flexibility [9, 11, 13]. Vegetation indices are based on combinations of spectral reflectance from different wavelengths representing the physiological status of the crop. Several studies have used UAV imagery-based vegetation indices as proxies to various plant growth parameters [12, 14–16], nitrogen content [17], and biotic [18, 19] and abiotic stresses [20, 21] with considerable success. Condorelli et al. [14] demonstrated a greater potential for UAV-based NDVI when compared to NDVI from a ground-based platform for monitoring wheat biomass accumulation and leaf greenness during its reproductive stage. A greater time requirement has been considered as one of the major concerns for the underperformance of ground-based platforms in that study, as rapid data acquisition is the key for high-throughput phenotyping.

As mentioned above, there are several studies that have used the UAV-based approach to monitor crop growth and health status and quite a few studies compared this approach with ground-based measurements. However, in the context of high-throughput phenotyping, we have found no studies that investigated whether visual ratings can be replaced by image-based vegetation indices. Therefore, the objective of this study is to determine if UAV imagery provides accurate and consistent estimations of crop injury from herbicide application and its potential as an alternative to visual ratings.

For this study, we used fababean (*Vicia faba* L.) as a test crop. Fababean is grown in Western Canada as a high protein feed and food crop. It has low competition with weeds in the seedling stage, and there are few registered herbicides. Therefore, in this study, fababean crop tolerance to nine herbicides or herbicide tank mixtures was evaluated.

## 2. Materials and Methods

### 2.1. Field Study

The study was conducted at Kernen Crop Research Farm, University of Saskatchewan, Saskatoon, SK, in 2016 and 2017. The soil at the study location was silty clay loam, with ~4.5% organic matter and a pH of ~7.5 in both years. Fertilizer application was followed based on the soil test recommendations.

The “CDC Snowdrop” variety of fababean was seeded in 2 × 6m plots at a rate of 40 seeds/m^2^, at a seeding depth of 3 cm, and with a row spacing of 30 cm. The plots were seeded on 13^th^ May and 5^th^ May in 2016 and 2017, respectively.

**Figure 1:**
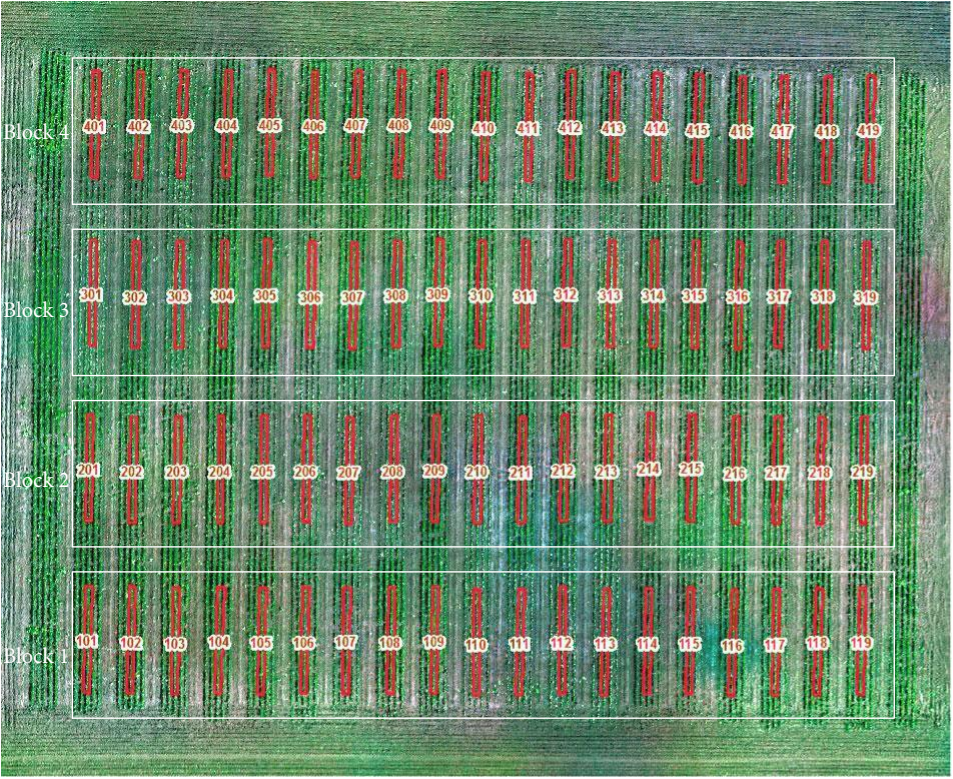
Field experimental layout of the study. 19 herbicide treatments were randomly distributed within each block; four white rectangles denote four blocks. The red rectangle covers a single middle row in each plot representing the polygon layer used for OSAVI calculation. The number in each plot represents the plot number.

The treatments include a hand-weeded check and 9 herbicide combinations with 2 rates (Table 1) distributed in a randomized complete block design (RCBD) with 4 blocks (Figure 1). The hand-weeded check was used to compare the relative efficacy of herbicide treatments. The treatments included herbicides that are not registered for use on fababean with the exception of bentazon; therefore, damage was observed on various plots. The chosen treatments were applied at the label rate and twice the label rate (Table 1). The herbicide treatments were applied on June 6^th^ (Biologische Bundesanstalt, Bundessortenamt, und CHemische Industrie (BBCH) stage code 16) in 2016 and June 12^th^ (BBCH stage code 34) in 2017 as a foliar spray. Visual ratings, growth reduction, and physiological chlorosis using a 0-100% visual rating scale where “0” represents no visual phytotoxicity and “100” represents complete plant mortality [22] were recorded by an experienced weed scientist on a whole plot basis, a week after the treatment application.

### 2.2. Image Acquisition

A multirotor quadcopter UAV called Draganflyer Commander (Draganfly Innovations Inc., Saskatoon, SK Canada) equipped with a MicaSense RedEdge 3 multispectral camera (MicaSense Inc., Seattle, WA, USA) was used for the image acquisition. Imaging has been done on the same day of visual ratings in both years. A mean temperature of 15-20°C and a wind speed of 10-20 km/h have been observed during image acquisition in both years.

The UAV has been programmed to fly the trial autonomously at a ground speed of 1.7 m/s (meters per second) and at 20 m AGL (above ground level). The altitude (20 m) was selected to obtain adequate ground sample distance (GSD) with a relatively low spatial resolution of the RedEdge camera. The images were captured from nadir view while maintaining 80% front and 80% side image overlap throughout the mission.

The focal length of the RedEdge camera is 5.5 mm, and the ground sample distance (GSD) of the camera at 20 m AGL was 13.6 mm. The RedEdge camera captures images in five spectral bands: blue (475 nm), green (560 nm), red (668 nm), red edge (717 nm), and near-infrared (840 nm). To accomplish this, the camera is equipped with 5 separate sensors which operate almost at the same time. Images of the RedEdge calibration target were obtained before and after the mission, and additionally, a DLS (downwelling light sensor) has been integrated with the RedEdge camera that measures the solar irradiance.

### 2.3. Image Processing

The UAV captured 264 (2016) and 325 (2017) images per band during the mission. The image processing software Pix4Dmapper Pro (Pix4D SA, 1015 Lausanne, Switzerland) was used to process the RAW imagery (16-bit TIF format). Pix4Dmapper workflow has 3 basic steps:

1. initial processing, that includes identification of key points (feature identification) from the overlapping images, matching, bundle block adjustment to reconstruct the position and orientation of each image, and calibration of lens distortion and other intrinsic parameters
2. point cloud densification and mesh generation, which help in the generation of a densified 3D point cloud from the sparse point cloud and construction of a 3D textured mesh based on the dense point cloud
3. generation of a digital surface model (DSM), orthomosaic, and reflectance maps

The average cloud coverage during the image acquisition is around 40% in both years. Therefore, in this study, both reflectance panel images with known reflectance values and DLS irradiance information from image EXIF data were utilized in the radiometric calibration. Details of the use of the calibrated reflectance panels and conversion of raw pixel values to absolute reflectance values can obtained in the MicaSense Knowledge Base (https://support.micasense.com/hc/en-us/articles/115000765514-Use-of-Calibrated-Reflectance-Panels-For-RedEdge-Data). The generated 5 reflectance maps, one each for all 5 bands, were further used in vegetation index calculations.

### 2.4. Vegetation Index (VI) Calculation

**Figure 2:**
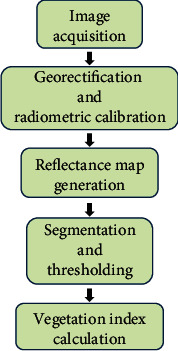
Image processing workflow for vegetation index calculation.

Esri ArcGIS 10.4.1 (Esri, Redlands, CA, USA) was used for plot segmentation, index calculation, and thresholding (Figure 2). In this study, instead of the whole plot, the single middle row was segmented to calculate the VI because of weed pressure within the plots. The “Create Feature” tool was used to create a polygon layer and to segment the crop rows from the reflectance maps. The “Raster Calculator” tool was used to calculate the vegetation index. For this study, the optimized soil-adjusted vegetation index (OSAVI) was calculated to compare against the visual ratings. OSAVI was selected because of its ability to minimize the effect of soil background brightness in the index calculations [23], particularly in relatively sparse vegetation conditions where the soil is visible through the canopy. OSAVI was calculated as follows:

(1) $$\begin{array}{c} \text{OSAVI}=\frac{R_{840}–R_{668}}{R_{840}+R_{668}+0.16}, \end{array}$$

where *R*_840_ and *R*_668_ are the reflectance values at bands centered on 840 (NIR) and 668 (red), respectively, and 0.16 is the soil adjustment coefficient.

**Figure 3:**
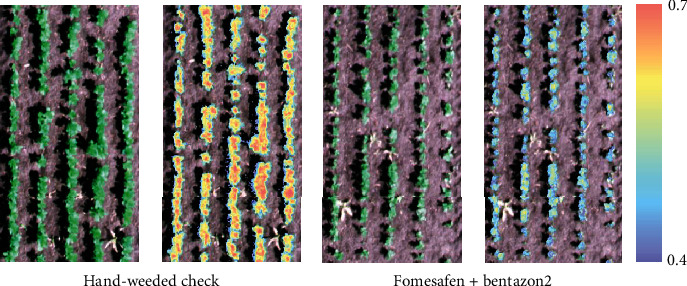
Sample RGB and OSAVI images of hand-weeded check and herbicide-treated (fomesafen+bentazon2) plots. The legend represents the variation of OSAVI values between hand-weeded check and herbicide-treated plots.

Thresholding was done to separate the crop vegetation pixels from the soil background, shadows, and weeds in the row. A threshold pixel value was selected by comparing the RGB and OSAVI maps. The thresholding value that included the maximum number of vegetation pixels without background noises (soil, weeds, and shadows) was decided. The conditional function (Con (,)) in the “Raster Calculator” tool was used to achieve the desired thresholding (Figure 3). Final mean index values per treatment were obtained using the “Zonal Statistics” tool in ArcGIS. The final index values from ArcGIS were adjusted based on the area of the thresholded vegetation pixels.

### 2.5. Statistical Analysis

Analysis of variance (ANOVA) was conducted, with herbicide as the main factor. Based on the year × fixedeffect interaction (*P* < 0.05), it has been decided to analyze the data separately for both the years and the analysis showed that the herbicide rate has no significant effect on plant tolerance (data not shown). When the ANOVA suggested a significant main effect, Tukey's honest significant difference (HSD) tests were used for mean separation. All ANOVA and mean separation calculations were performed using PROC GLM in SAS (SAS version 9.4, SAS Institute Inc., Cary, NC). PROC CORR in SAS was used to calculate correlations between visual ratings and vegetation indices.

## 3. Results and Discussion

### 3.1. Herbicide Treatments

**Figure 4:**
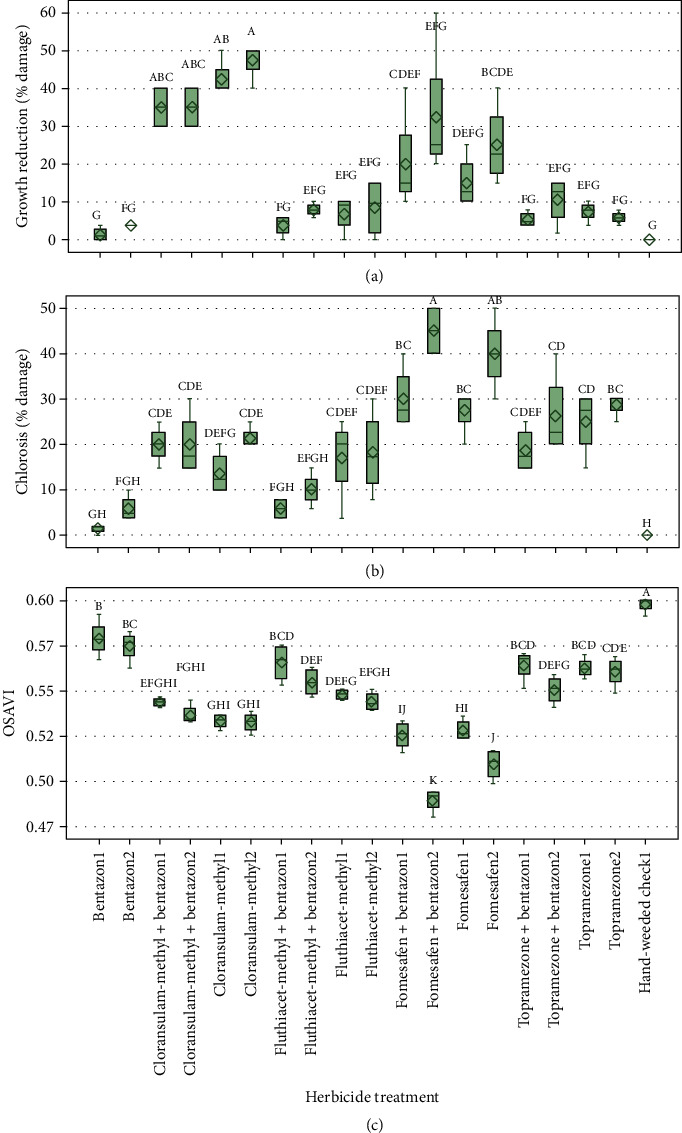
2016 ANOVA results of fababean. (a) Physiological growth reduction (%). (b) Physiological chlorosis (%). (c) OSAVI (optimized soil-adjusted vegetation index) values for all the herbicide treatments and rates. Herbicide name followed by “1” denotes single dose and name followed by “2” denotes double dose. Error bars represent the standard error of differences between the means. Comparisons were made between the treatments; means followed by the same letter are not significantly different at *P* < 0.05.

**Figure 5:**
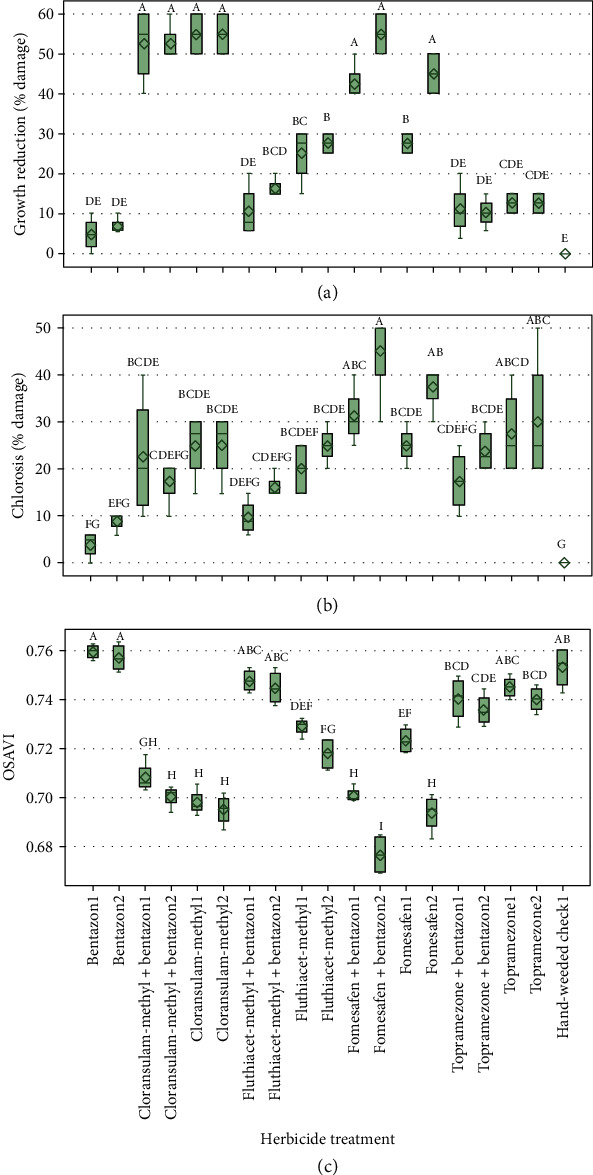
2017 ANOVA results of fababean. (a) Physiological growth reduction (%). (b) Physiological chlorosis (%). (c) OSAVI (optimized-soil adjusted vegetation index) values for all the herbicide treatments and rates. Herbicide name followed by “1” denotes single dose and name followed by “2” denotes double dose. Error bars represent the standard error of differences between the means. Comparisons were made between the treatments; means followed by the same letter are not significantly different at *P* < 0.05.

In both years, bentazon herbicide was well tolerated by fababean as there is no significant damage to the crop compared to the untreated hand-weeded check with corresponding higher OSAVI values (Figures 4 and 5). This is not surprising as bentazon is registered in fababean and was included in the study as an industry standard check. Bentazon combined with other herbicides showed mixed results (Figures 4 and 5). Cloransulam-methyl and fomesafen applied alone and in a tank-mix combination with bentazon resulted in higher plant growth reduction and lower vegetation index values than the other herbicide treatments, including the untreated hand-weeded check (Figures 4 and 5). In contrast, physiological chlorosis ratings were relatively low compared to growth reduction for these treatments. The variable injury symptoms arising from herbicides with different mechanisms of action makes the visual assessments more difficult and increases the subjectivity factor.

### 3.2. Phenotypic Precision

Overall, the UAV imagery-based vegetation index (OSAVI) produced more precise results compared to visual ratings for both years (Figures 4 and 5). The significantly better coefficient of variation (CV) values were observed for OSAVI when compared to visual ratings (Table 2). Respective CV values for OSAVI, visual growth reduction, and visual chlorosis in 2016 and 2017 were 1.29 and 0.79%, 42.65 and 17.88%, and 25.64 and 31.66% (Table 2). The coefficient of variation denotes the experimental error as a percentage of the mean and can be used to compare the precision of different assessment methods or raters [24]. Similar results were reported by Guan and Nutter [25], who compared the visual assessment method (percent defoliation) and proximal sensing method (percent reflectance) to quantify the alfalfa (*Medicago sativa* L.) foliar pathosystem. Results from this study showed higher precision for the percent reflectance method with a standard error of estimate and CV values considerably lower than the percent defoliation method.

The higher CV values of visual herbicide damage ratings in this study (Table 2) suggest a low level of repeatability among the replications when compared to the UAV-based approach. This lack of consistency is because of the intrarater variability, which is defined as the variability between the repeated assessments on the same sampling unit made by the same rater or instrument [25]. Furthermore, greater repeatability among the replications reduces standard error of *y*-estimate leading to improved statistical separation among the treatment means [25]. Similarly, it was observed in this study that Tukey's HSD test yielded more precise mean separation for the UAV-based vegetation index than visual ratings (Figures 4 and 2). For example, in 2016, OSAVI values of hand-weeded check1 treatment were found to be significantly different from other treatments; however, that was not the case in the manual methods (Figures 3 and 4). In 2017, fomesafen+bentazon2 treatment showed greater crop damage with a corresponding significantly lower OSAVI value compared to other treatments, whereas, such separation of this treatment was not observed in visual assessments (Figure 5). Manual methods often suffer from low repeatability, and there are several factors such as rater-related, plant-related, and damage/symptom-related that can be attributed to the errors and subjectivity of visual assessments in the field conditions [26]. Furthermore, in some cases, it may not be possible for the naked eye to identify plant physiological/metabolic differences due to different stresses [27], but it may be possible with imaging techniques [28].

In contrast to the manual methods, remotely sensed vegetation indices are less biased and more precise compared to visual rating methods especially for canopy-scale measurements [25]. Similar to the results of this study, Nutter et al. [24] reported a greater precision in the estimation of bentgrass (*Agrostis palustris* Huds.) dollar spot damage for the spectral reflectance method than the visual observation method. In a study to screen the drought-adaptive traits in wheat, Condorelli et al. [14] reported higher repeatability for UAV-based NDVI measurements compared to the ground-based greenseeker sensors. The study suggested that with the level of precision of UAV-based high-throughput phenotyping, identification of QTLs for drought-adaptive traits is possible.

In the context of high-throughput phenotyping, where large trials are more common and raters are expected to rate thousands of plots, manual assessments introduce issues related to rater's efficiency, including physical tiredness due to rating for a long period, greater time requirement inducing plant stage variability, and error associated with intra- and intervariability from the use of multiple raters [24]. In the present study, some of these issues were avoided as the total number of plots rated in this study was <80 and the rating has been done by a single rater and simultaneously with the aerial imaging.

One of the key aspects of the UAV-based platform is the high-throughput capability. In our study, although the total number of plots rated were low (<80), we found noticeable time requirement differences between the methods. The UAV-based method with adequate spatial resolution (ground sample distance 1.2 cm) took 1/10 of the time required for the visual assessment of the plots. Furthermore, in large phenotyping projects, the time requirements will be substantially high for the visual rating method compared to the UAV-based platforms. Similar results were also reported by Nutter and Guan (2003), where the manual defoliation method took 15 times more time than the remote-sensing method to estimate alfalfa foliar disease intensity. The time component is a prerequisite for high-throughput phenotyping. Longer time requirements for assessments may introduce variability due to changes in plant stage, environmental conditions, and rater capability that can affect the repeatability and lead to inaccurate scores and misinterpretations [7, 14].

Despite the differences in the consistency, we observed stronger correlations of OSAVI with visual ratings for both years (Table 3). Particularly, visual growth reduction seems to have a greater correlation with the vegetation index. This suggests that image-based measurements follow similar trends as visual ratings but with improved precision. Jansen et al. [29] explored the use the noninvasive spectral phenotyping methods in *Cercospora* disease resistance screening in sugar beet. Strong correlations between vegetation indices and visual scores in that study suggested the potential for the use of spectral methods in disease resistance breeding.

It was observed that the correlations were slightly lower between growth reduction and OSAVI in 2016 compared to 2017 (Table 3) and slightly higher index values for the year 2017 (Figure 5) compared to 2016 (Figure 4). This is probably associated with the better maintenance of weed-free conditions in the plots in 2017 reducing the need for aggressive thresholding during image processing and index generation.

## 4. Conclusions

The study was primarily designed to compare the phenotypic precision of both digital and visual rating methods. Therefore, the conclusions made in this study should be applied cautiously while comparing other methods. Lower CVs and standard errors of OSAVI in this study suggest that undesirable variability associated with visual assessments can be minimized with the UAV-based approach. The lack of repeatability between the replications is the main reason for the poor performance of the visual rating method. In the context of high-throughput phenotyping, which requires the assessment of a large number of plants in a short period of time, consistent performance of the rater/raters will be an issue leading to inaccurate assessments. Furthermore, repeatability is often associated with heritability in breeding programs, hence measurements with low repeatability are generally removed from the analysis [27]. This increases the need for more replications and locations to attain adequate repeatability/heritability which may lead to additional cost to the breeding programs. As per the results of this study, UAV-based methods with a high-throughput capability and adequate precision have the potential to replace visual ratings, especially for canopy-scale measurements.

## Figures and Tables

**Table 1:** List of herbicide and rate treatments.

Trt no.	Treatment name	^∗^Rate (g a.i. ha^−1^)
1	Hand-weeded check	—
2	Topramazone	12.5
3	Topramazone	25
4	Bentazon	475
5	Bentazon	950
6	Fomesafen	140
7	Fomesafen	280
8	Topramazone+bentazon	12.5 + 475
9	Topramazone+bentazon	25 + 950
10	Cloransulam-methyl	17
11	Cloransulam-methyl	34
12	Cloransulam-methyl+bentazon	17 + 475
13	Cloransulam-methyl+bentazon	34 + 950
14	Fomesafen+bentazon	140 + 840
15	Fomesafen+bentazon	280 + 1683
16	Fluthiacet-methyl	4
17	Fluthiacet-methyl	8
18	Fluthiacet-methyl+bentazon	4 + 475
19	Fluthiacet-methyl+bentazon	8 + 950

^∗^g a.i. ha^−1^: grams active ingredient per hectare.

**Table 2:** Coefficient of variation (CV) and mean of the visual ratings and vegetation index for 2016 and 2017.

Parameter	CV		Mean	
	2016	2017	2016	2017
Visual growth reduction	42.65	17.88	16.57	27.50
Visual chlorosis	25.64	31.66	19.76	21.65
OSAVI	1.29	0.79	0.54	0.72

**Table 3:** Correlation coefficients of OSAVI and visual ratings for the years 2016 and 2017.

	2016		2017	
	Visual growth reduction	Visual chlorosis	Visual growth reduction	Visual chlorosis
OSAVI	-0.63785<0.0001	-0.76883<0.0001	-0.91724<0.0001	-0.67355<0.0001
